# 11′-Deoxyverticillin A (C42) promotes autophagy through K-Ras/GSK3 signaling pathway in HCT116 cells

**DOI:** 10.1007/s13238-014-0099-z

**Published:** 2014-09-29

**Authors:** Shubin Niu, Dongdong Yuan, Xuejun Jiang, Yongsheng Che

**Affiliations:** 1 State Key Laboratory of Mycology, Institute of Microbiology, Chinese Academy of Sciences, Beijing, 100101 China; 2 State Key Laboratory of Toxicology & Medical Countermeasures, Beijing Institute of Pharmacology & Toxicology, Beijing, 100850 China; 3 University of Chinese Academy of Sciences, Beijing, 100039 China; 4 Center for New Drug Evaluation, Shandong Academy of Pharmacology, Jinan, 250101 China

**Dear Editor**

Macroautophagy (hereafter referred to as autophagy) is a cellular adaptation to starvation, involving the vesicular sequestration of the long-lived cytoplasmic proteins and organelles such as mitochondria (Klionsky and Emr, 2000). However, it also results in cell death when overactivated (Edinger and Thompson, 2004). Stresses which affect cellular homeostasis including changes in oxidation status, build-up of damaged organelles and misfolded proteins, and infection by pathogens, have also been shown to induce autophagy (Rabinowitz and White, 2010). Besides, autophagy was found to be involved in cancer formation and progression. Depending on tumor type, stage, and cellular context, autophagy can play either anti- or pro-tumorigenic roles (Mancias and Kimmelman, 2011).

11′-Deoxyverticillin A (C42), a natural product isolated from the *Cordyceps*-colonizing fungus *Gliocladium* sp., is a member of a class of fungal secondary metabolites known as epipolythiodioxopiperazines (ETPs) (Chen et al., 2009). ETPs were reported to have a broad range of biological activities including antitumor effect (Chen et al., 2005). In previous study, we have first demonstrated that the C42-induced autophagy preceded caspase-dependent apoptosis in HCT116 cells and could independently lead to cell death, in addition to association with apoptotic cell death (Zhang et al., 2011b), but the mechanism of this process remained to be unexplored.

Electron microscopy, which is believed to be one of the most convincing instruments to detect autophagy, was used to investigate the autophagy induced by C42 (Klionsky et al., 2012). Compared to the control, an obvious accumulation of membrane vacuoles was found in the C42-treated HCT116 cells and cytosolic components or organelles were sequestered in some of the vacuoles. Autophagosome-like vacuoles with double-membrane structures (high magnification) and a segment of the double-membrane between a vacuole and mitochondrion were also observed (Fig. 1A).

**Figure. 1:**
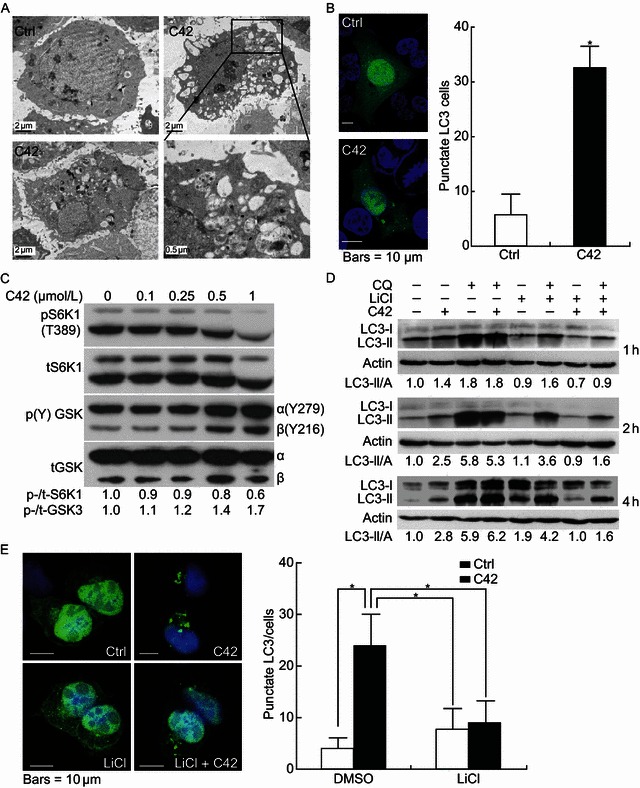
**The C42 enhances autophagy via GSK3 signaling pathway**. (A) Electron microscopy was performed on vehicle (ctrl) and the C42-treated (0.5 μmol/L, 3 h) HCT116 cells as described in Materials and Methods. The right picture of the lower row indicated the high-contrast image of the cell region marked by the box. (B and E) HCT116 cells were transfected with a plasmid expressing GFP-LC3. After 12 h, the cells were treated with the presence or absence of LiCl for 3 h at 37°C in RPMI-1640 medium with 1/1000 DMSO (Ctrl), and C42 (0.5 μmol/L). Following fixation, the cells were stained with DAPI and visualized immediately by fluorescence microscopy. The number of punctuate GFP-LC3 in each cell was counted, and at least ten cells were included in each group. The data were normally distributed and were statistically analyzed. The asterisks denote a significant difference between the groups (*P* < 0.01). (C) HCT116 cells were treated with increasing concentrations of C42 (0.1–1.0 μmol/L) for 1 h, harvested, lysed, and immunoblotted for indicated proteins. The levels of p-p70S6 K (S6K1, Thr389) and p-GSK (Y216/279) were detected by Western blot analysis. (D) HCT116 cells were treated with C42 (0.5 μmol/L) in the presence or absence of LiCl and chloroquine (CQ) for up to 4 h before analysis by immunoblotting with the indicated antibodies. The lysates were analyzed by Western blot with the antibodies indicated. Densitometry was performed for quantification. The adjusted ratios of LC3-II to actin were calculated and presented below the blots. The ratios represent the results of three independent experiments

To further measure autophagosome formation in the C42-treated cells, HCT116 cells were transfected with a fusion protein of green fluorescent protein (GFP) and LC3 (a specific marker of autophagosomes) and visualized by confocal microscopy (Klionsky et al., 2012). While GFP-LC3 staining remained diffused in the control cells, C42 challenge resulted in an increased punctate staining of GFP-LC3 (Fig. 1B) (*P* < 0.01), suggesting that C42 increases the formation of autophagosome.

Since the ratio of LC3-II to actin is an accurate indicator of autophagy, the expression of LC3-II in HCT116 cells was detected following the treatment of C42. As shown in Fig. S1A, the ratio of LC3-II to actin relative to the controls was increased after C42 challenged for 1 and 3 h, respectively. SQSTM1/p62, which is considered to be a selective substrate of autophagy, was decreased when treated with C42 (Fig. S1A), indicating that C42 induces autophagy in the cells. To detect autophagic flux, the level of LC3-II was measured in the presence of bafilomycin A1 (Baf A1), which acts as a specific inhibitor of the vacuolar type H^+^-ATPase in cells, thus blocking acidification of organelles and subsequent fusion of the autophagosome with the lysosome (Klionsky et al., 2012). Baf A1 addition resulted in further accumulation of LC3-II in the cells tested (Fig. S1B), confirming the aforementioned results that C42 activates autophagic process.

Mammalian target of rapamycin (mTOR) inhibits autophagy and its kinase activity can be detected by measuring the phosphorylation of its substrates, such as p70S6 kinase (S6K1). As shown in Figs. 1C and S2, treatment of HCT116 by C42 attenuated the phosphorylation of S6K1 in a dose-dependent manner, suggesting that the agent induces autophagy by inhibiting the mTOR/S6K1 pathway.

Interestingly, we found that C42 promoted autophagy concomitantly with increase in Tyr279/216 phosphorylation of GSK3 at both the 1 and 3 h time points (Figs. 1C and S2), indicating that GSK3 may participate in the C42-induced autophagy.

To verify the involvement of GSK3 in the C42-induced autophagy, the inhibitors of GSK3, LiCl and SB216763, were used in the following detection. As expected, the presentation of either LiCl or SB216763 reduced the LC3-II accumulation (Figs. S3A and S3B) and GSK3 phosphorylation at Tyr279/216 caused by C42 (Fig. S3C). Moreover, addition of chloroquine (CQ), an agent often used in autophagic flux detection (Klionsky et al., 2012), failed to accumulate LC3-II when the cells were challenged with both C42 and LiCl at the indicated time points (Fig. 1D), implying that inhibition of GSK3 blocks the C42-dependent autophagy. Besides, using confocal microscope, we observed that LiCl decreased the formation of autophagosome induced by C42 (Fig. 1E).

We performed gene knockdown using specific siRNA against GSK3 to further examine its role in the C42-promoted autophagy. In contrast to the Mock-control, silencing either GSK3α (Fig. S4A) or GSK3β (Fig. S4B) or both (Fig. 2A) inhibited LC3-II accumulation induced by C42.

**Figure. 2:**
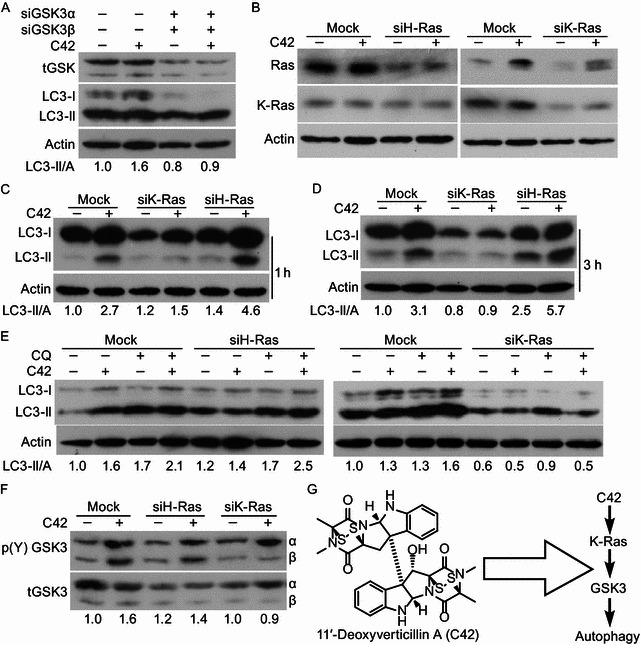
**K-Ras silencing blocks the C42-induced LC3-II accumulation concurring with a decrease in the phosphorylation of GSK3 at Y216/279**. HCT116 cells were transfected with the control (Mock), GSK3α + β (A), H-Ras and K-Ras (B–E) siRNA. After 48 h of transfection, cells were treated with C42 (0.5 μmol/L) in the absence (A, B, C, D and F) or presence (E) of chloroquine (CQ) for up to 3 h (A, B, C, E and F: 1 h, D: 3 h). The lysates were analyzed by Western blot with the antibodies indicated. Densitometry was performed for quantification. The adjusted ratios of LC3-II to actin and phosphorylated- to total GSK3 were calculated and presented below the blots. The ratios represent the results of three independent experiments. G Schematic representation of the signaling pathways through which C42 induced autophagic process

Since mutation of K-Ras is one of the most frequent genetic events in human cancer, with the highest incidences in colorectal tumors (30%–60%) (Brink et al., 2003) and either oncogenic H-Ras or K-Ras is reported to regulate autophagy in different conditions (Marino et al., 2011), we explored whether they played a role in the C42-induced autophagy. We use specific siRNA to down-regulate the mRNA level of H- or K-Ras in HCT116 cells (Fig. 2B). Interestingly, K-Ras silencing remarkably decreased LC3-II accumulation upon stimulation with C42, whereas the compound was found to increase LC3-II levels in the H-Ras-depleted cells (Fig. 2C and 2D). Unlike H-Ras depleted cells, CQ and C42 failed to accumulate LC3-II in the K-Ras-depleted cells (Fig. 2E), indicating that K-Ras, not H-Ras, plays a critical role in regulating the C42-dependent autophagy.

Meanwhile, a study has shown that K-Ras is essential for GSK3 activation, which played a critical role in regulating autophagy (Zhang et al., 2011a). Therefore, we examined the status of GSK3 following the Ras depletion. Notably, the deprivation of K-Ras, but not H-Ras, blocked the C42-induced phosphorylation of GSK3 at Tyr279/216 (Fig. 2F), suggesting that C42 enhanced autophagy via K-Ras/GSK3 signaling pathway (Fig. 2G).

The crosstalk between Ras and autophagy is highly complex and widely studied. It has been reported that overexpression of K-Ras G12V in NIH3T3 fibroblasts inhibited the starvation-dependent autophagy (Furuta et al., 2004). Another study revealed that H-Ras G12V blocked autophagy induced by matrix-detachment in non-malignant rat intestinal epithelial line IEC-18 cells (Yoo et al., 2010). In the present study, we clearly showed that K-Ras, not H-Ras, plays an essential role in mediating the C42-induced autophagy. The findings here are consistent with the conception that Ras mediated autophagy depending on cell type. Moreover, we speculated that the Ras-promoted autophagy was likely to be stimulus type- and time-dependent.

Interestingly, we observed that GSK3 also functioned as a downstream signaling molecular of K-Ras to regulate the C42-induced autophagy. Recently, GSK3 was reported to be required in regulation of autophagy. In one work, autophagy was activated through the GSK3-Tip60-ULK1 signaling pathway (Lin et al., 2012). Moreover, cadmium was found to promote autophagy through the ROS-GSK3β signaling pathway (Wang et al., 2009). Consistent with aforementioned studies, we found that GSK3 was further activated at Tyr279/216 in the C42-treated cells accompanied by accumulation of LC3-II, indicating that C42 also induce autophagy through the GSK3 dependent pathway.

The new finding in the present study is that K-Ras, not H-Ras, acts upstream of GSK3 to mediate the C42-induced autophagy. Since both Ras and GSK3 signaling pathways are well known targets for cancer therapy, we believe that the results from this study will provide information for development of therapeutics targeting Ras and GSK3 by controlling autophagy in cancer treatment.

## FOOTNOTES

We thank Dr. Tamotsu Yoshimori from Osaka University for the GFP-LC3 plasmid. This work was supported by grants from the National Natural Science Foundation of China (Grant Nos. 31171329 and 31070149), Beijing Natural Science Foundation (5111003), and the National Program of Drug Research and Development (2012ZX09301-003).

S. N. and D. Y. performed the experiments; Y. C. and X. J. designed the experiments.

Shubin Niu, Dongdong Yuan, Xuejun Jiang and Yongsheng Che declare that they have no conflict of interest. This article does not contain any studies with human or animal subjects performed by the any of the authors.

## Electronic supplementary material

Below is the link to the electronic supplementary material.

Supplementary material 1 (PDF 229 kb)
