# Supplementary material for: 11′-Deoxyverticillin A (C42) promotes autophagy through K-Ras/GSK3 signaling pathway in HCT116 cells
Source: Protein Cell. 2014 Sep 29;5(12):945–9. doi: 10.1007/s13238-014-0099-z (PMC4259883; doi:10.1007/s13238-014-0099-z)
Supplement: Supplementary file 1 — Supplementary material 1 (PDF 229 kb) [file 13238_2014_99_MOESM1_ESM.pdf]

SUPPLEMENTARY MATERIALS

Materials and Methods

Chemicals and antibodies

11'-Deoxyverticillin A was isolated from the solid-substrate fermentation culture of the *Cordyceps*-colonizing fungus *Gliocladium* sp. (Chen et al., 2009). The reagents, SB216763 (S3442), LiCl (L9650), chloroquine (C6628), and polyclonal antibodies against LC3 (L7543), were purchased from Sigma-Aldrich. The antibodies to phospho-p70S6K (Thr389; 9205), p70S6K (9202), and Ras (3965), were from Cell Signaling Technology, and those to actin (sc-1616-R) and SQSTM1/p62 (sc-28359) were purchased from Santa Cruz Biotechnology. The antibodies for Phospho-GSK3α/β (Y279/Y216; 2309-1), GSK3α (2309-1), GSK3(C-term; 1561-1), and K-Ras (ab55391) were purchased from Epitomics (Abcam).

Fungal material and extracts preparation

The strain of the *Cordyceps*-colonizing fungus was isolated by Prof. Xingzhong Liu (Institute of Microbiology, Chinese Academy of Sciences) from the samples of *Cordyceps sinesis* (Berk.) Sacc. collected from Linzhi, Tibet, in March, 2004. The fungus was identified as *Gliocladium* sp. and assigned the accession No. XZC04-CC-302 in X.L.'s culture collection at the Institute of Microbiology, Chinese Academy of Sciences, Beijing. The crude extract was prepared as described previously (Liu et al., 2008).

Plasmids and siRNAs

The GFP-LC3 plasmid was a gift from Dr. Tamotsu Yoshimori (Osaka University,

Japan). The siRNA specific for human GSK3α (SC-29339), GSK3β (sc-35527), H-Ras (SC-29340), and K-Ras (SC-35731), were purchased from Santa Cruz Biotechnology along with the control siRNA.

Cell culture and Western blot analysis

Human colon carcinoma cells (HCT116) were grown in RPMI-1640-medium (HyClone, SH30809. 01B) with 10% FBS and antibiotics. Cells were split overnight and grown to 50% confluence before adding 11'-deoxyverticillin A (C42). For siRNA interference, cells were grown to 30% confluence in their respective medium without antibiotics and transfected using DharmaFECT (Dharmacon, T2001) according to the manufacturer's instructions. After transfection for 48 h, cells were directly treated with C42 without being split. Whole cell lysates were prepared with lysis using Triton X-100/glycerol buffer, which contained 50 mM Tris-HCl, pH 7.4, 2 mM EDTA, 2 mM EGTA, and 1 mM dithiothreitol, supplemented with 1% Triton X-100 and protease inhibitors. The lysates were then separated on a SDS-PAGE gel (15 or 8% according to the molecular weights of the proteins of interest) and transferred to PVDF membrane. Western blotting was performed using appropriate primary antibodies and horseradish peroxidase-conjugated secondary antibodies, followed by detection with enhanced chemiluminescence (Pierce Chemical, 34080).

Confocal microscopy

HCT116 cells were transfected with the GFP-LC3 expressing plasmid and after 24 h, cells were treated with C42, GFP-LC3 fluorescence was imaged, and the images were acquired via confocal microscopy (Leica, TCS SP5).

Electron microscopy

Electron microscopy was performed as described previously (Li et al., 2008). Briefly, HCT116 cells were washed three times with PBS, trypsinized and collected by centrifugation. The cell pellets were fixed with 4% paraformaldehyde overnight at 4 °C, post-fixed with 1% OsO_4 in cacodylate buffer at room temperature for 1h and dehydrated stepwise with ethanol. The dehydrated pellets were rinsed with propylene oxide for 30 min and then embedded in Spurr resin for sectioning. Images of thin sections were observed under a transmission electron microscope (JEM1230, Tokyo, Japan)

Statistical analysis

Normally distributed data are shown as mean ± SD and were analyzed using one-way analysis of variance and the Student-Newman-Keuls post-hoc test.

Supplementary Figures

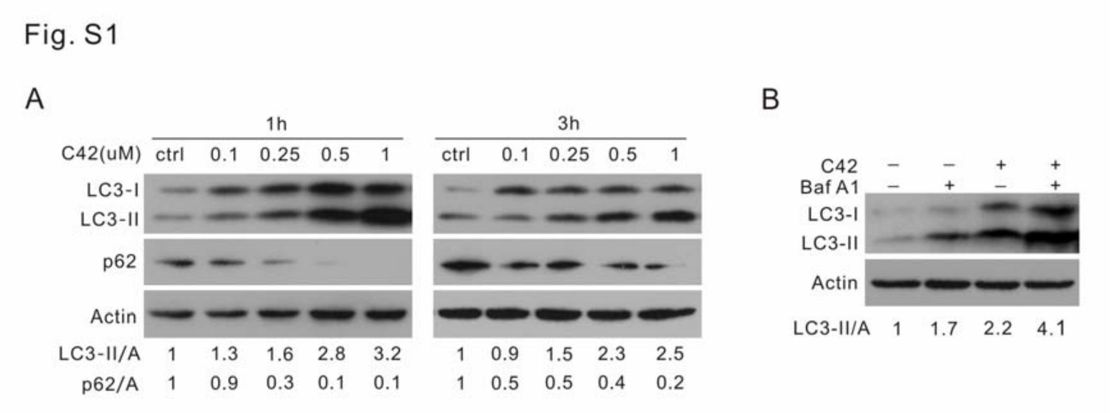

Figure S1. C42 induces LC3-II accumulation and enhances autophagic flux. (A) HCT116 cells were treated with C42 (0.1–1.0 μM) for up to 3 h, harvested, lysed, and immunoblotted for indicated proteins, and then detected by Western blot analysis.

Densitometry was performed for quantification and the ratios of LC3-II and SQSTM1/p62 to actin were calculated and presented below the blots. (B) HCT116 cells were treated with C42 (0.5 μM) in the presence of bafilomycin A1 (BafA1, 20 μM). Densitometry was performed for quantification. The ratios of LC3-II and SQSTM1/p62 to actin were presented below the blot. All data were acquired from at least three independent experiments.

Fig. S2

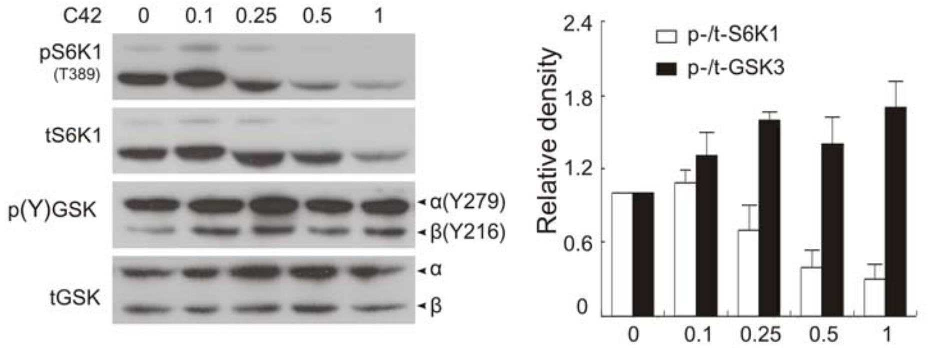

Figure S2. C42 decreases p70S6K phosphorylation concurring with increased activity of GSK3. HCT116 cells were treated with increasing concentrations of C42 (0.1–1.0 μM) for 3 h, harvested, lysed, and immunoblotted for indicated proteins. The levels of p-p70S6K (S6K1, Thr389) and p-GSK (Y216/279) were detected by Western blot analysis. Densitometry was performed for quantification and the ratios of the phophorylated- to total p70S6K and GSK3 were presented in the graphs on the right side. All data were acquired from at least three independent experiments.

Fig. S3

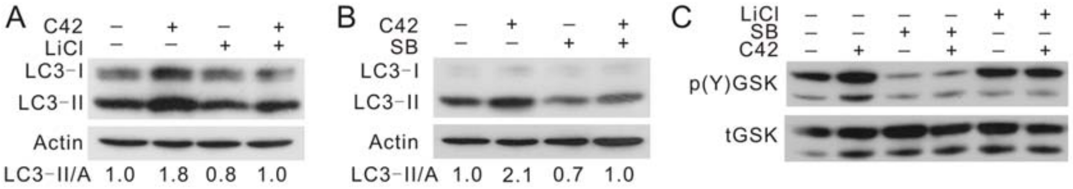

Figure S3. The C42 enhances autophagy is GSK3-dependent. HCT116 cells were treated with C42 (0.5 μM) in the presence or absence of LiCl (A and C) and SB216763 (B and C) for 1 h before analysis by immunoblotting with the indicated antibodies. The lysates were analyzed by Western blot with the antibodies indicated. Densitometry was performed for quantification. The adjusted ratios of LC3-II to actin were calculated based on the data obtained and presented below the blots. The ratios represent the results of three independent experiments.

Fig. S4

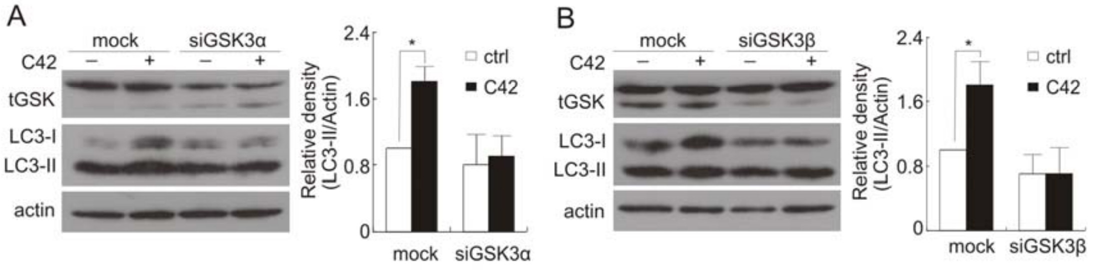

Figure S4. K-Ras silencing blocks the C42-induced LC3-II accumulation. HCT116 cells were transfected with the control (Mock), GSK3*α* (A), GSK3*β* (B) After 48 h of transfection, cells were treated with C42 (0.5 μM) for 3 h before immunoblotting analysis with the indicated antibodies. Densitometry was performed for quantification and the ratios of LC3-II to actin were presented in the graph on the right side. All data were acquired from at least three independent experiments. The

asterisks denote a significant difference between the groups ($p < 0.05$).

Supplementary References

Chen, Y., Guo, H., Du, Z., Liu, X.Z., Che, Y., and Ye, X. (2009). Ecology-based screen identifies new metabolites from a Cordyceps-colonizing fungus as cancer cell proliferation inhibitors and apoptosis inducers. Cell Proliferat 42, 838-847.

Li, M., Jiang, X., Liu, D., Na, Y., Gao, G.F., and Xi, Z. (2008). Autophagy protects LNCaP cells under androgen deprivation conditions. Autophagy 4, 54-60.

Liu, L., Liu, S., Jiang, L., Chen, X., Guo, L., and Che, Y. (2008). Chloropupukeananin, the first chlorinated pupukeanane derivative, and its precursors from Pestalotiopsis fici. Org Lett 10, 1397-1400.
